# Economic Evaluation of Transcatheter Aortic Valve Replacement Compared to Surgical Aortic Valve Replacement in Chinese Intermediate-Risk Patients

**DOI:** 10.3389/fcvm.2022.896062

**Published:** 2022-05-26

**Authors:** Weicong Zhang, Yake Lou, Yujiang Liu, Hongwei Wang, Chun Zhang, Linxue Qian

**Affiliations:** ^1^Department of Ultrasound, Beijing Friendship Hospital, Capital Medical University, Beijing, China; ^2^Department of Cardiology, The Second Affiliated Hospital of Chongqing Medical University, Chongqing, China; ^3^Department of Radiology, Dongzhimen Hospital Affiliated to Beijing University of Chinese Medicine, Beijing, China; ^4^Department of Ultrasound, Beijing Anzhen Hospital, Capital Medical University, Beijing, China

**Keywords:** TAVR, SAVR, economic evaluation, cost-effectiveness, aortic stenosis

## Abstract

**Background:**

Aortic stenosis (AS) is a severe disease that causes heart failure and sudden death. Transcatheter aortic valve replacement (TAVR) and surgical aortic valve replacement (SAVR) are both recommended for patients with intermediate surgical risk, but the cost-effectiveness of TAVR compared to SAVR in China has not been investigated.

**Methods:**

A combined decision tree and Markov model were conducted to compare the cost-effectiveness of TAVR versus SAVR with a 5-year simulation. The primary outcome was the incremental cost-effectiveness ratio (ICER), a ratio of incremental costs to incremental quality-adjusted life-year (QALY). One-way sensitive analysis and probabilistic sensitivity analysis (PSA) were conducted to test the robustness of the model.

**Results:**

After a simulation of 5 years, the costs of TAVR and SAVR were 54,573 and 35,002 USD, respectively, and the corresponding effectiveness was 2.826 versus 2.712 QALY, respectively. The ICER for the TAVR versus SAVR comparison was 170,056 USD/QALY, which was three times higher than the per capita gross domestic product (GDP) in China. One-way sensitive analysis showed that the cost of the TAVR device impacted the ICER. The TAVR could be cost-effective only in the case where its cost is lowered to 29,766 USD.

**Conclusion:**

TAVR is currently not cost-effective in China, but it could be cost-effective with a reduction of costs to 29,766 USD, which is approximately 65% of the current price.

## Introduction

Aortic stenosis (AS) is a severe disease that causes heart failure and sudden death (1, 2). A retrospective survey conducted in China showed that the prevalence of AS was 0.16% in inpatients younger than 65 years old and that it was 0.41 and 0.56% in those aged 65–74 and over 75 years old (3). Another study conducted in China found that 0.39–0.66% of outpatients aged over 65 years who received echocardiography were diagnosed with severe AS (4). As the Chinese population is entering an aging society, the burden of AS is increasing.

Surgical aortic valve replacement (SAVR) has always been the optimal treatment for patients with AS across different risk stratifications (5, 6). However, several important clinical studies regarding transcatheter aortic valve replacement (TAVR) (7–10) have demonstrated the efficacy and safety of TAVR all over the world (11, 12). It is estimated that more than 306,000 patients with AS have undergone TAVR in the United States (13). The number of patients AS who have undergone TAVR in China is much lower but is increasing at a fast rate.

In patients with AS who are at intermediate risk for surgery, it has been demonstrated that TAVR has similar efficacy as that of SAVR (9). The 2021 European Society of Cardiology (ESC) guidelines for valvular heart disease recommended that SAVR and TAVR are both first-line treatments for patients at intermediate-risk (6). In clinical practice, whether a treatment can be widely used depends not only on its effectiveness but also on whether it is cost-effective. The collective purchase policy launched by the Chinese govenment allows only cost-effective drugs or medical devices to be widely used in Chinese hospitals, but an economic evaluation comparing TAVR versus SAVR is lacking. Thus, the present study aimed to investigate the cost-effectiveness of TAVR compared to SAVR among Chinese patients at intermediate-risk.

## Materials and Methods

### Overview

The basic structure of the model consisted of two parts, namely, a 30-day decision tree and a 59-month Markov model. Patients who entered the model would first enter the decision tree, and a TAVR or SAVR was performed. After 30 days, the patients would enter a Markov model with a simulation of 59 months. The summary simulation period was 60 months. The starting age was 80 years old, and the simulation cycle was 5 years, which was similar to that in the PARTNER 2 study.

### Model Structure

**FIGURE 1:**
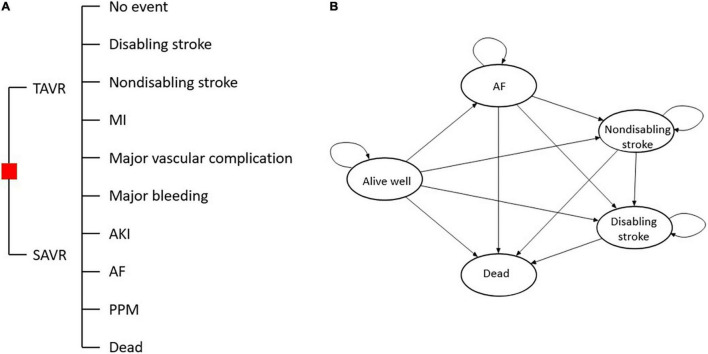
Decision tree **(A)** and state transition diagram of the Markov model **(B)**.

In the 30-day decision tree model, patients allocated to the TAVR or SAVR group may experience one or several complications of the procedure, including death, disabling stroke, non-disabling stroke, myocardial infarction (MI), major vascular complication, major bleeding, acute kidney injury (AKI), permanent pacemaker implantation, and new atrial fibrillation (AF). After that, the patients would enter a Markov model with a simulation of 59 months, and every patient entering the Markov model would transit among five states, including no events, post-AF, post-disabling stroke, post-non-disabling stroke, and death. The cycle period in the Markov model was 1 month, and there were 59 cycles in summary. The detailed model is displayed in Figure 1.

### Input Parameters

#### Clinical Data

The clinical data analyzed in our study was mainly derived from the PARTNER 2 study (Placement of aortic transcatheter valves II - XT intermediate and high risk) (9, 14). For periprocedural complications within 30 days, the corresponding data were directly extracted from a published article. For data between 1 month and 5 years post-procedure, they were transformed into probability per month. Considering that the probabilities of complications and death within 1, 2, and 5 years may vary, we separately calculated the data between these periods. The non-cardiovascular mortality in the Markov model was obtained from the China National Bureau of Statistics^1^. As the AF incidence was much higher in patients with a procedure than without a procedure, the AF incidence was accessed from the PARTNER 2 study. However, the mortality of AF was obtained from a study conducted in a Chinese population (15), and the mortality of stroke was also derived from a Chinese cohort study. The key input parameters in this study are listed in Table 1.

**TABLE 1:** Periprocedural complications incidence and transition probabilities in the model.

	Base	*SD*	Range low	Range high	Source
**Periprocedural complications incidence in TAVR (30 days)**					
AF	0.091	0.009	0.073	0.109	(14)
AKI	0.013	0.004	0.006	0.02	(14)
Bleeding	0.104	0.01	0.085	0.123	(14)
Death	0.039	0.006	0.027	0.051	(14)
Disabling stroke	0.032	0.006	0.021	0.043	(14)
Major vascular complication	0.079	0.008	0.062	0.096	(14)
MI	0.012	0.003	0.005	0.019	(14)
Non-disabling stroke	0.023	0.005	0.014	0.032	(14)
PPM	0.085	0.009	0.068	0.102	(14)
**Periprocedural incidence in SAVR (30 days)**					
AF	0.264	0.014	0.237	0.291	(14)
AKI	0.031	0.005	0.02	0.042	(14)
Bleeding	0.434	0.016	0.404	0.464	(14)
Death	0.041	0.006	0.029	0.053	(14)
Disabling stroke	0.043	0.006	0.031	0.055	(14)
Major vascular complication	0.05	0.007	0.037	0.063	(14)
MI	0.019	0.004	0.011	0.027	(14)
Non-disabling stroke	0.018	0.004	0.01	0.026	(14)
PPM	0.069	0.008	0.053	0.085	(14)
**Transition probabilities of no event to AF in TAVR (per month)**					
2–12 months	0.001	/	/	/	(14)
13–24 months	0.0011	/	/	/	(14)
25–60 months	0.0014	/	/	/	(9)
**Transition probabilities of no event to AF in SAVR (per month)**					
2–12 months	0.001	/	/	/	(14)
13–24 months	0.0001	/	/	/	(14)
25–60 months	0.0011	/	/	/	(9)
**Transition probabilities of no event to non-disabling stroke in TAVR (per month)**					
2–12 months	0.0007	/	/	/	(14)
13–24 months	0.0003	/	/	/	(14)
25–60 months	0.0005	/	/	/	(9)
**Transition probabilities of no event to non-disabling stroke in SAVR (per month)**					
2–12 months	0.0007	/	/	/	(14)
13–24 months	0.0003	/	/	/	(14)
25–60 months	0.0003	/	/	/	(9)
**Transition probabilities of no event to disabling stroke in TAVR (per month)**					
2–12 months	0.0017	/	/	/	(14)
13–24 months	0.0011	/	/	/	(14)
25–60 months	0.0011	/	/	/	(9)
**Transition probabilities of no event to disabling stroke in SAVR (per month)**					
2–12 months	0.0014	/	/	/	(14)
13–24 months	0.0005	/	/	/	(14)
25–60 months	0.0007	/	/	/	(9)
**Cardiovascular mortality in TAVR (per month)**					
2–12 months	0.0036	/	/	/	(14)
13–24 months	0.0027	/	/	/	(14)
25–60 months	0.0067	/	/	/	(9)
**Cardiovascular mortality in SAVR (per month)**					
2–12 months	0.0047	/	/	/	(14)
13–24 months	0.0029	/	/	/	(14)
25 and 60 months	0.0057	/	/	/	(9)
Non-cardiovascular mortality for aged 80–85 (per month)	0.0026	/	/	/	(15)
Transition probability of AF to stroke (per month)	0.0016	/	/	/	(28)
Transition probability of AF to disabling stroke (per month)	0.0011	/	/	/	(28)
Transition probability of AF to non-disabling stroke (per month)	0.0005	/	/	/	(28)
Transition probability of AF to death (per month)	0.0024	/	/	/	(28)

#### Costs

The key costs are displayed in Table 1. The costs of TAVR are shown in USD, including the TAVR device costs, medicine costs, diagnosis costs, and other costs, and the overall costs of SAVR are also shown in USD. Different from previous studies, the costs in the present study were derived from a domestic article, and the costs of intensive care unit (ICU) or ward stay were covered in the medicine and other costs. The costs of stroke, AF, MI, major bleeding, AKI, and permanent pacemaker were obtained from a published article. Because there are no explicit costs of major vascular complications, we consulted two experts in this field and adopted the value of 5,000 USD as its cost. All the costs were discounted at.037 annually, which was the mean medical consumer price index (CPI) in the past 5 years in China. The range of costs was extracted from a published article. If the costs could not be extracted from a published article, we adopted 0.5 fold and 2 fold as the lower and higher limits, respectively. All the costs were converted from Chinese renminbi (RMB) to USD at a ratio of 6.5, which was the mean ratio in 2021.

#### Utilities

If there were utilities for the Chinese population, we adopted the domestic value; otherwise, we adopted the commonly used utilities. The base utilities of post-procedure were obtained from a published article investigating the utilities of TAVR and SAVR, and we adopted the disutility for complications including AF, bleeding, major vascular complications, non-disabling stroke, and AKI. The utility for disabling stroke was a fixed value of.39, which is commonly used in published studies.

### Outcomes

The primary outcome of this study was the incremental cost-effectiveness ratio (ICER), representing the incremental costs per quality-adjusted life-year (QALY). As there was no specific willing-to-pay (WTP) threshold in China, we selected three times the per capita GDP in China in 2021 as the WTP, which was 37,500 USD. The TAVR would be considered cost-effective if the ICER was less than 37,500 USD/QALY; otherwise, it would be thought as not cost-effective. In addition, if the TAVR was not cost-effective, the cost leading to cost-effectiveness would be calculated.

### Sensitive Analysis

One-way sensitive analysis was conducted to compare the effects of variables on ICER, and the result was illustrated in a tornado diagram. Probabilistic sensitive analysis (PSA) was employed using 10,000 times of Monte Carlo simulation. All the costs were assumed to follow the gamma distribution, and probabilities were assumed to follow the beta distribution. Cost-effectiveness acceptability curves and a scatter plot were used to show the uncertainty.

Given that the cost of the TAVR device may vary among different regions, we also adopted different costs for scenario analysis.

## Results

The periprocedural complication incidence, transition probabilities, utilities, and costs are listed in Tables 1, 2. The range, distribution, and sources are also displayed in Tables 1, 2. All the analyses were performed based on the above data.

**TABLE 2:** Utilities and costs in the model.

Utility	Base	*SD*	Range low	Range high	Sources
No event in TAVR <7 months	0.74	0.24	/	/	(19, 22)
No event in TAVR 7–12 months	0.76	0.2	/	/	(19, 22)
No event in TAVR >12 months	0.75	0.22	/	/	(19, 22)
No event in SAVR <7 months	0.68	0.24	/	/	(19, 22)
No event in SAVR 7–12 months	0.75	0.27	/	/	(19, 22)
No event in SAVR >12 months	0.74	0.23	/	/	(19, 22)
Disabling stroke	0.39	/	0.31	0.52	(29)
**Disutility**					
Non-disabling stroke	–0.161	0.054	/	/	(22)
AF	–0.038	/	–0.038	0	(24)
AKI	–0.177	/	–0.177	0	(24)
Bleeding	–0.447	/	–0.447	0	(24)
Major vascular complication	–0.046	/	–0.046	0	(24)
Myocardial infarction	–0.1	/	–0.1	0	(30)
**Costs**					
TAVR device	45526	11511	22965	68087	(21)
TAVR diagnosis	2016	721	1008	4031	(21)
TAVR medicine	2025	1163	1013	4050	(21)
TAVR others	824	112	605	1043	(21)
SAVR device	15580	15933	7790	31160	(21)
SAVR diagnosis	2076	677	749	3403	(21)
SAVR medicine	8182	5703	4091	16364	(21)
SAVR others	1401	1883	700	2801	(21)
Non-disabling stroke event	1898	/	1096	2390	(31)
Non-disabling annual cost	1349	329	404	1721	(31)
Disabling stroke event	2509	/	1379	3291	(31)
Disabling stroke annual cost	2053	516	516	2582	(31)
Myocardial infarction event	6750	/	3375	13500	(32)
Major vascular complication	5500	/	2750	11000	Calculation
Major bleeding	868	69	732	1003	(33)
AKI	1849	1176	924	3697	(34)
New permanent pacemaker	13680	4380	5094	22265	(35)
AF event	16192	/	14124	18475	(36)
AF annual cost	1891	/	945	3781	(37)
Stroke death	2151	458	1011	2843	(31)
Discount rate	0.037	/	/	/	(38)

### Base Case

In the base case, after a simulation of 5 years, compared to SAVR, TAVR gained plus.115 QALY (2.826 vs. 2.712 QALY) but led to a higher cost of 54,573 USD, which was 35,002 USD in SAVR. The ICER of TAVR versus SAVR was 170,056 USD/QALY, which was higher than three times the per capita GDP in China (Table 3).

**TABLE 3:** Base case and scenario analysis based on different TAVR device cost.

	Arm	TAVR/SAVR costs (USD)	Summary Costs (USD)	Summary Effectiveness (QALY)	Incremental Cost (USD)	Incremental Effectiveness (QALY)	ICER (USD/QALY)
Base case	SAVR	15580	35001	2.71	/	/	/
	TAVR	45526	54573	2.83	19571	0.115	170056
Scenario 1	TAVR	33846	43266	2.83	8265	0.115	71813
Scenario 2	TAVR	17268	27219	2.83	–7782	0.115	–67621
Scenario 3	TAVR	26794	36439	2.83	1438	0.115	12500
Scenario 4	TAVR	29766	39316	2.83	4315	0.115	37500

### Sensitive Analysis

**FIGURE 2:**
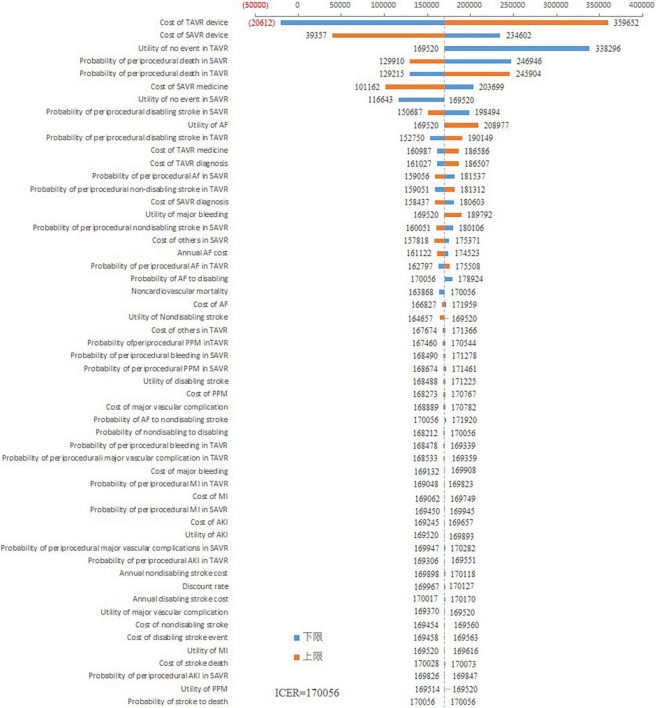
Tornado diagram based on the one-way sensitivity analysis.

As shown in Figure 2, the cost of the TAVR device had the greatest impact on ICER. When the cost of the TAVR device fluctuated from 22,965 to 68,086 USD, the ICER ranged from –20,611 to 359,652 USD/QALY. The costs of the SAVR device also impacted the ICER. The ICER fluctuated between 39,357 and 234,602 USD/QALY when the SAVR cost decreased from 31,159 to 7,790 USD. Other variables had little impact on the ICER fluctuation. The ICER was consistently greater than 100,000 USD/QALY regardless of the changes in other variables.

**FIGURE 3:**
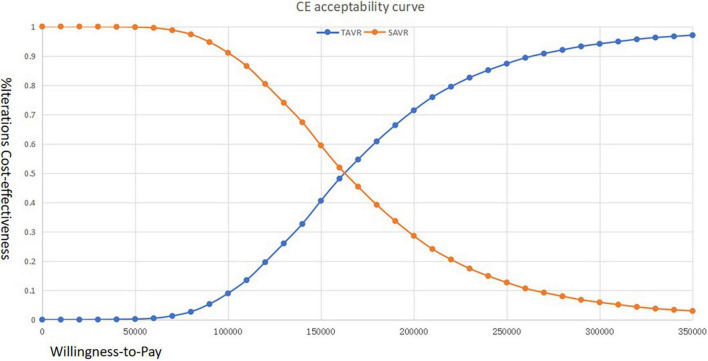
Cost-effectiveness acceptability curve of transcatheter aortic valve replacement (TAVR) vs. surgical aortic valve replacement (SAVR) among Chinese patients with aortic stenosis (AS) who are at immediate risk.

**FIGURE 4:**
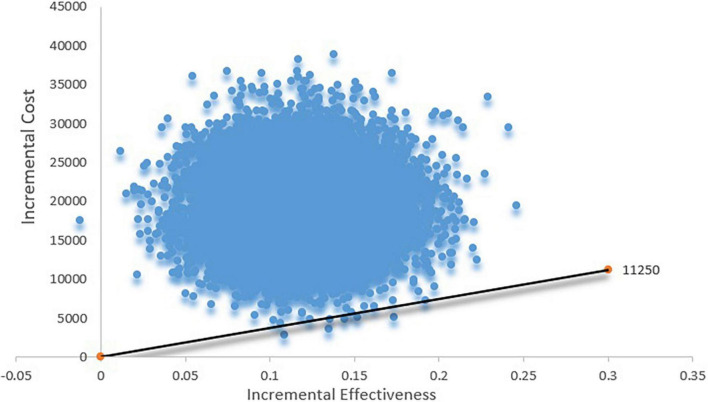
Scatter plot based on probabilistic sensitive analysis. The probability that TAVR is cost-effective is less than 5%.

The cost-effectiveness acceptability curve suggested that when the WTP threshold is 100,000 USD, the acceptability of TAVR is < 10%. If the WTP is > 160,000 USD, the acceptability can exceed 50% (Figure 3). The scatter plot indicated that under the current context, TAVR could be cost-effective with a 5% probability (Figure 4).

### Scenario Analysis

The current price of the imported TAVR device is approximately 45,526 USD, ranging from 17,268 to 40,975 USD in regions outside China, and the price for the domestic TAVR device is approximately 33,846 USD. We performed a scenario analysis based on the above costs and found that under the current Chinese domestic TAVR device costs, the TAVR is still not cost-effective, and the ICER is 71,813, which is 5.75 times higher than the current per capita GDP in China. However, if we adopted the price of TAVR in Canada, TAVR would be cost-effective due to its lower costs compared to SAVR. In addition, we also found that when the TAVR price is 26,794 USD, the ICER would be 12,500 USD/QALY, which is equal to the current per capita GDP in China, and the ICER would be 37,500 USD/QALY (three times greater than the current per capita GDP in China) when the TAVR price is 29,766 USD.

## Discussion

To the best of our knowledge, the present study is the first to investigate the cost-effectiveness of TAVR vs. SAVR in Chinese patients with AS. We found that in the intermediate surgical risk population, TAVR is not currently cost-effective in China, and TAVR could be cost-effective only when the TAVR device cost is decreased to 29,766 USD. Thus, if the TAVR device cost is lowered to 26,794 USD, TAVR would be highly cost-effective.

Some studies have reported that TAVR is cost-effective in their countries (16–19). However, in the present study, we concluded that TAVR is not currently cost-effective in China due to several reasons. First, the costs of the TAVR device vary among different regions, ranging from 17,268 USD to 45,526 USD (20, 21). In Canada, the cost is 17,268 USD, but the cost is approximately 45,526 USD in China, resulting in an ICER of 170,056 USD/QALY, which is higher than the per capita GDP in China. However, if we adopted the Canadian TAVR device cost in our analysis, the cost of TAVR would be less but the effect would not change. Second, as China is the largest developing country, the per capita GDP is only 12,500 USD, which is lower than that in the United States, Canada, Australia, and Japan, which may cause a lower threshold of WTP. The ICER of 170,056 USD/QALY may be accepted in the United States and Australia with a per capita GDP of more than 60,000 USD, but it cannot be currently accepted in China. Third, there is a difference in the composition of surgical costs between China and other countries. In China, the TAVR device costs account for over 90% of the overall costs, and the proportion of device costs is much higher in China than in other countries (21). In the USA, the proportion of the TAVR device costs is less than 65% of the total costs, and in Australia and Canada (20, 22), the proportion of the TAVR device costs is lower than 60%. The unique situation in China leads to the fact that TAVR is not currently cost-effective in China.

Compared to SAVR, TAVR achieves similar clinical outcomes (7–9). In inoperative patients, TAVR may significantly reduce mortality and other outcomes (23), but in patients with high risk or intermediate risk, the published clinical trials have indicated that TAVR displays similar efficacy to that of SAVR (7, 14). The improvement of TAVR versus SAVR may lie in the relief of symptoms, indicating that patients who underwent TAVR may achieve higher utilities than those who received SAVR, especially in the periprocedural period (24). In the PARTNER 2 study, the periprocedural mortality is 6.1% in the TAVR group versus 8.0% in the SAVR group, but these differences are not statistically significant. When the follow-up period is extended to 5 years, the mortalities in TAVR and SAVR groups are 47.9 and 43.4%, respectively (9, 14). A previous meta-analysis conducted by our team has also demonstrated that TAVR has similar efficacy to that of SAVR regardless of the follow-up period (10). A similar efficacy but higher costs may suggest that TAVR is not cost-effective in China. In addition, the durability of TAVR should be investigated. However, studies thus far have shown that TAVR is safe. The durability of SAVR needs to be evaluated for at least 10 years of follow-up (25), but the longest reported follow-up period of TAVR is only 6 years (26).

One-way sensitive analysis showed that the costs of the TAVR device had the largest impact on the ICER. The PSA showed that TAVR was cost-effective only with a 5% probability. These results indicated that under current costs, TAVR is not cost-effective. The scenario analysis showed that when the costs of the TAVR device are decreased to 29,766 USD, the TAVR could be cost-effective. If the costs of TAVR are lowered to 26,794 USD, TAVR would be highly cost-effective. The Chinese government has launched a collective purchase project, which requires that only cost-effective drugs or medical devices can be used in public hospitals in China, indicating that only drugs or medical devices listed in the collective purchase can be widely used in China at present (27). The present study demonstrated that TAVR could be cost-effective only when the costs are lowered to 29,766 USD. Importantly, the present study provided a viewpoint for TAVR from the Chinese health care system payer’s perspective.

The present study had several limitations. First, the cardiovascular mortality in our study was derived from the PARTNER 2 study with only a few Chinese patients included in the study. The cardiovascular mortality in Chinese patients who underwent TAVR may be slightly different from that in the PARTNER 2 study. Second, the simulation period in our study was 5 years, which was consistent with the PARTNER 2 study, but some studies have shown that a longer follow-up period may allow the TAVR to be cost-effective. Third, the data in our study were transformed from a published article. The inability to access the raw data limited our further analysis. Last, the present study was performed based on a published study rather than real-world data in China. Thus, real patient-level data may be more appropriate, indicating that additional studies based on the Chinese population are needed.

## Conclusion

Transcatheter aortic valve replacement is not currently cost-effective in China. However, TAVR could be cost-effective with a reduction of costs to 29,766 USD, which is approximately 65% of the current price.

## Data Availability Statement

The original contributions presented in the study are included in the article/supplementary material, further inquiries can be directed to the corresponding author/s.

## Author Contributions

LQ and CZ came up with the idea and designed the protocol. WZ and YL synthesized the data and drafted the manuscript. YL and HW participated in the data collection and data analysis. All authors approved the final version of the manuscript.

## Conflict of Interest

The authors declare that the research was conducted in the absence of any commercial or financial relationships that could be construed as a potential conflict of interest.

## Publisher’s Note

All claims expressed in this article are solely those of the authors and do not necessarily represent those of their affiliated organizations, or those of the publisher, the editors and the reviewers. Any product that may be evaluated in this article, or claim that may be made by its manufacturer, is not guaranteed or endorsed by the publisher.

## Footnotes

1
http://www.stats.gov.cn/
